# Exploring the relationship between YouTube video characteristics and a viewer’s mental health traits among young adults

**DOI:** 10.3389/fpsyt.2024.1364930

**Published:** 2024-07-05

**Authors:** Go Eun Choi, Miran Pyun, So-Hee Yoon, Yeongchae Kim, Hyejin Shin, Sang Yup Lee

**Affiliations:** ^1^ Department of Digital Analytics, Yonsei University, Seoul, Republic of Korea; ^2^ Department of Communication, Yonsei University, Seoul, Republic of Korea

**Keywords:** YouTube, violence, brightness and saturation, mental health, deep learning

## Abstract

We investigated the relationship between individuals’ mental health traits and the characteristics of YouTube videos they watch. The mental health traits considered were stress, depression, anxiety, and self-esteem, which were measured using a survey questionnaire. We considered violence shown in a video, brightness and saturation of a video as video characteristics. We utilized the viewing history log data of the participants and analyzed the videos they watched on YouTube using computer vision techniques based on deep learning algorithms. The results revealed that viewers’ consumption of violent videos was positively related to stress, depression, and anxiety, but negatively related to self-esteem. Individuals with higher levels of stress, depression, or anxiety tended to view darker videos than those with lower levels of stress, depression, or anxiety.

## 1 Introduction

YouTube is the largest video-sharing platform in the world and the second most popular social media platform after Facebook (1). According to a report published by eMarketer in 2023 (2), the average time spent on YouTube per day in 2022 was approximately 52 minutes, a 30 percent increase from 2019 prior to COVID-19, when it was 39.7 minutes. Teens and youth tend to spend more time on YouTube than other age groups. For example, according to the Pew Research Center (3), approximately one in five teenagers in the United States visit or use YouTube almost constantly.

With the popularity of YouTube and the large amount of time spent on it, several scholars have raised concerns about its potentially problematic use and negative effects on users’ mental health (4, 5). In this regard, various studies have examined the relationship between viewers’ mental health and YouTube use (e.g., 6, 7).

However, most of these studies have limitations. One of the limitations is the fact that prior studies placed their primary focus on the time spent on YouTube or addictive YouTube use and examined how it is associated with an individual’s mental health (e.g., 7, 8). Although it is necessary to investigate the relationship between mental health and the time spent on YouTube, it is equally important to correctly understand the relationship between viewers’ mental health traits and the characteristics of YouTube videos they consume, a relationship that has received little attention.

If there is a systematic relationship between viewers’ mental health characteristics and the features of YouTube videos watched, then information about those features can be used to indirectly diagnose or monitor their mental health status. In other words, information about YouTube videos can be utilized to create digital phenotyping profiles that show viewers’ mental health status.

Multiple studies have found that content uploaded to social media platforms, such as Instagram and Twitter, reflects a user’s mental health (e.g., 9–13). Specifically, these studies analyzed photos and text uploaded to social media platforms and found that the features of the posts varied according to the mental health characteristics of the user. For example, individuals with depression tend to post darker photos on Instagram (10) and use words associated with negative emotions and anger more frequently (11). However, to the best of our knowledge, no studies have examined the relationship between the characteristics of content consumed on social media, particularly YouTube, and users’ mental health traits.

Therefore, examining the relationship between YouTube video characteristics and mental health traits can help us better understand the connection between mental health and social media use. Moreover, if a systematic relationship between YouTube video characteristics and mental health traits is discovered, it can be combined with information obtained from other social media platforms that a person uses, which is likely to result in more accurate information about a person’s mental health status.

Another limitation of previous studies examining the relationship between mental health and YouTube use is that they primarily relied on surveys to measure individuals’ YouTube use (e.g., 7). Respondents’ survey answers are based on memory; hence, the answers may have lacked accuracy. In this regard, log data of a user’s viewing history can more accurately assess YouTube use in terms of the amount of time spent on YouTube and the types of videos viewed.

To bridge this gap in the literature, this study investigates the relationship between an individual’s mental health traits and the characteristics of the videos they watch on YouTube. The mental health traits considered in this study were stress, depression, anxiety, and self-esteem, which were measured using a survey questionnaire. For the characteristics of YouTube videos, we considered the violence, brightness and saturation of a video. We utilized the viewing history log data of the participants and analyzed the videos they watched on YouTube using computer vision techniques based on deep learning algorithms. Specifically, we propose the following research questions:

RQ1) What is the relationship between the level of violence in YouTube videos and the mental health characteristics of viewers?

RQ2) What is the relationship between the brightness of YouTube videos and the mental health characteristics of viewers?

RQ3) What is the relationship between the saturation of YouTube videos and the mental health characteristics of viewers?

## 2 Methods

### 2.1 Data

#### 2.1.1 Log data on viewing history on YouTube

From September 10 to October 31, 2022, forty-five individuals, who reported having a YouTube account and routinely viewing videos on the site visited our research laboratory to provide log data of their YouTube viewing history. In the lab, the participants logged into their YouTube accounts and downloaded viewing logs between June 1 and August 31, 2022, using Google Takeout, which they shared with the authors. Owing to unidentified technical issues, the data of eight of the forty-five participants were not completely downloaded and were excluded from the final dataset. The log data included the URLs, dates, and titles of videos viewed by the participants. The study included a total of 37 individuals, consisting of 28 females and nine males. The average age of the participants was 23.51 years, with a median age of 23 and a standard deviation of 1.94.

#### 2.1.2 Institutional review board approval statement

This study was approved by the IRB of Yonsei University, South Korea (No. 7001988-202209-HR-1672-02).

#### 2.1.3 Video data

We downloaded the video files and additional information about each video, such as its category and length, for further analysis using URLs. To download the video files, we used the “PyTube” library provided in Python, a programming language.

### 2.2 Measures

#### 2.2.1 Violence in a video

To measure the violence depicted in a YouTube video, we extracted an image from the video every three seconds. Subsequently, we employed supervised learning approaches based on deep learning computer vision techniques to identify whether each of the extracted images was violent. To implement the supervised image classification task, the authors constructed a training dataset using three different publicly available violence-related datasets: the XD-Violence dataset,^1^ Video Fight Detection dataset,^2^ and Real Life Violence Situations dataset.^3^ The datasets contained inaccurate or unidentifiable images;^4^ therefore, we only incorporated images from those datasets that were identified as violent or non-violent by three human coders unanimously to construct a more accurate training dataset. An image was identified as violent if it involved scenes of 1) attacking or harming another person or animal using a part of the human body (e.g., hand, foot, etc.) or an object (e.g., weapon, knife, chair, brick, etc.) or 2) something being destroyed. These guidelines were based on a study by Williams et al. (14).

The final training dataset consisted of 5,400 violent and 5,400 non-violent images. We used a pre-trained RegNet model proposed by the Facebook research team for image classification (15) because this model outperformed state-of-the-art models, such as ResNet, ResNexT, and EfficientNet. For the test dataset, the accuracy of the model was 0.8767. To validate the performance of the trained model with respect to the YouTube video dataset used in this study, we randomly selected 100 images from our dataset, applied the trained model, and obtained an accuracy of .85.

We applied the trained model to each image extracted from the video and obtained the probability that the image was violent. To determine the likelihood of a video being violent, we used the following equation:


pv=∑i=1Kpv,iK

where $p_{v}$ is the probability that video v is violent, K is the number of video frames extracted from video v , and $p_{v,i}$ is the probability that frame. is violent as calculated using the trained RegNet model.

#### 2.2.2 Brightness and saturation of a video

To determine the brightness and saturation of the color in the video, we used the OpenCV library provided in Python. We first extracted an image from a video every three seconds and calculated the brightness and saturation of each image using the OpenCV library. To determine the brightness and saturation of the video, we used the average brightness and saturation of the images extracted from the video. The average brightness of the video was calculated as follows (a similar equation was used to calculate the average saturation of the video):


Bv=∑i=1KBv,iK

where $B_{v}$ is the brightness of the color of video v , K is the number of video frames extracted from video v , and is the brightness of the color of image which was calculated using the OpenCV library.

#### 2.2.3 Mental health traits

After providing the researchers with YouTube log data, the participants completed a questionnaire to assess their mental health status. The questionnaire measured depression, generalized anxiety, stress, social phobia, social comparison, and self-esteem. Even though the following scales have been commonly employed in prior studies to measure mental health traits, it is important to acknowledge that the questionnaire’s results are subjective. This implies that the responses provided may not accurately represent the mental health traits of the respondents. Prior to responding to the survey questionnaire, the participants were given a concise explanation by the trained research assistants regarding the study’s objective and the survey’s process. Respondents conducted the survey within a specifically assigned laboratory.

Depression was measured using the Center for Epidemiological Studies Depression Scale (16), which was composed of 20 times. Example items include “I feel depressed” and “I thought my life was a failure.” The answer options range from 1 (seldom) to 4 (almost always). The value of Cronbach alpha was 0.86.

Generalized anxiety was measured using the 7-item Generalized Anxiety Disorder Scale (17). Example items are “I can’t stop or control my worrying” and “I get annoyed or angry easily.” The 5-point Likert scale was used, ranging from 1 (strongly disagree) to 5 (strongly agree). The value of Cronbach alpha was 0.87.

Stress was assessed with the Perceived Stress Scale developed by Cohen et al. (18), which consists of 10 items. Example items are “How often have you experienced feeling sensitive and stressed?” and “How often have you felt that you were unable to handle things you absolutely had to do?” The 5-point Likert scale was used, ranging from 1 (strongly disagree) to 5 (strongly agree). The Cronbach alpha value was 0.84.

Self-esteem was measured by the scale developed by Rosenberg in 1965 (19). The scale contained 8 items. Example items include “I am generally satisfied with myself” and “I don’t have much to be proud of.” The value of Cronbach alpha was 0.89.

### 2.3 Analyses

#### 2.3.1 Statistical models

As the main purpose of this study was to examine the association between mental health characteristics and YouTube video features, we used the following regression model (Equation 1):


(1) $$y_{i}=b_{0}+b_{1}X_{k,i}+\theta Z_{i}+u_{i}$$

where $y_{i}$ is the dependent variable of user i representing the YouTube video feature of interest, $X_{k,i}$ is the variable representing user i ‘s mental health trait k including depression, stress, anxiety, and self-esteem, is a set of control variables, including gender and age, and is an error term. The demographic factors (i.e., gender and age) have been considered as control variables mainly because they are likely to influence a viewer’s video choice. For example, male and younger viewers may have a greater inclination towards watching action-oriented videos compared to female and elderly viewers.

#### 2.3.2 Statistical estimation

The parameters in Equation 1 were estimated using different methods, depending on the type of the dependent variable. When the dependent variable was a ratio (e.g., the probability of a video being violent), we applied a fractional logit model and a Beta regression model, whereas for dependent variables that took values within limited boundaries (e.g., the brightness and saturation of video colors), we used Tobit regression models (20).

## 3 Results

### 3.1 Correlation coefficients and descriptive results

The correlation coefficients between mental health variables and variables representing YouTube video characteristics are presented in **Table 1**. The mean, median, and standard deviation values are also reported. Cronbach’s alpha values were reported for the mental health variables. The average violence level and saturation of a video were positively correlated to negative mental health traits such as stress and depression, while they were negatively correlated to the positive mental health trait, which is self-esteem. In contrast, the brightness of a video was negatively correlated to negative mental health traits, but positively correlated to self-esteem.

**Table 1:** Correlation coefficients and descriptive results.

	1	2	3	4	5	6	7	8	9
1. Stress	–								
2. Depression	0.73^**^	–							
3. Anxiety	0.56^**^	0.72^**^	–						
4. Self-esteem	−0.27	−0.43^**^	−0.26	–					
5. Avg. num of videos/month	0.04	0.16	−0.03	−0.08	–				
6. Avg. length of a video (minutes)	0.33^*^	0.20	0.10	0.07	−0.04	–			
7. Avg. violence of a video (%)	0.22	0.24	0.12	−0.27	0.02	−0.25	–		
8. Avg. brightness	−0.24	−0.20	−0.16	0.27	0.16	0.14	−0.35^*^	–	
9. Avg. saturation	0.13	0.11	0.18	−0.30	−0.03	0.05	0.47^**^	−0.46^**^	–
Mean	3	1.78	1.68	2.99	417.16	19.68	32.87	126.43	78.27
Median	3.2	1.75	1.43	2.9	306.66	15.37	32.97	129.22	78.49
S.D.	0.71	0.41	0.59	0.52	460.13	11.97	8.13	11.17	9.86
Cronbach’s alpha	0.84	0.86	0.87	0.89	–	–	–	–	–

N = 37, ^*^ p< 0.05, ^**^ p< 0.01.

### 3.2 Relationship between the amount of violent videos and mental health traits

The results of the relationship between the amount of violent videos and mental health traits are reported in **Table 2**. In the table, the association between each mental health trait and the average violence likelihood of videos, controlling for the effects of the viewer’s age and gender, is reported. The results in **Table 2** show positive associations between violence and mental health issues, whereas the relationship between violence and self-esteem was negative. The coefficients of stress and depression variables were statistically significant at a .05 significance level.

**Table 2:** Associations between violent videos and mental health traits (dependent variable: the violence of videos).

Variable	Coef.	S.E.	Variable	Coef.	S.E.	Variable	Coef.	S.E.	Variable	Coef.	S.E.
Female	−0.37	0.13	Female	−0.33	0.13	Female	−0.31	0.12	Female	−0.27	0.13
Age	−0.02	0.03	Age	−0.02	0.03	Age	−0.01	0.03	Age	−0.01	0.03
Stress	0.17^*^	0.07	Depression	0.28^*^	0.13	Anxiety	0.09	0.08	Self-esteem	−0.14	0.09
Constant	−0.49	0.64	Constant	−0.41	0.62	Constant	−0.37	0.74	Constant	0.24	0.81

N = 37, ^*^ p< 0.05.

### 3.3 Brightness and saturation

#### 3.3.1 Brightness

**Table 3** presents the estimation results of Equation 1 with respect to video brightness. The results in **Table 3** show that the associations between brightness of videos and negative mental health conditions (i.e., stress, depression, and anxiety) were negative; however, all the coefficients of the mental health-related variables were statistically insignificant at a .05 significance level.

**Table 3:** Estimation results of Equation 1 with respect to brightness (dependent variable: the brightness of videos).

Variable	Coef.	S.E.	Variable	Coef.	S.E.	Variable	Coef.	S.E.	Variable	Coef.	S.E.
Female	5.64	4.59	Female	4.43	4.62	Female	4.03	4.42	Female	2.68	4.96
Age	−0.17	0.84	Age	−0.10	0.88	Age	−0.30	0.82	Age	−0.17	0.81
Stress	−4.42	2.58	Depression	−5.48	3.89	Anxiety	−2.81	3.05	Self-esteem	5.14	3.08
Constant	138.68^**^	18.20	Constant	135.27^**^	19.33	Constant	135.11^**^	19.73	Constant	113.10^**^	24.36

N = 37, ^**^ p< 0.01.

#### 3.3.2 Saturation

The estimation results of Equation 1 with respect to video saturation are presented in **Table 4**. The results indicate that there were positive associations between video saturation and viewers’ negative mental health conditions, whereas the association between saturation and self-esteem was negative and only statistically significant.

**Table 4:** Estimation results of Equation 1 with respect to saturation (dependent variable: the saturation of videos).

Variable	Coef.	S.E.	Variable	Coef.	S.E.	Variable	Coef.	S.E.	Variable	Coef.	S.E.
Female	−7.96	4.30	Female	−7.11	4.20	Female	−6.92	3.90	Female	−5.55	4.10
Age	−0.37	0.72	Age	−0.39	0.79	Age	−0.36	0.70	Age	−0.44	0.67
Stress	3.06	1.89	Depression	3.74	2.98	Anxiety	3.37	2.99	Self-esteem	−5.11^*^	2.45
Constant	83.81^**^	15.57	Constant	86.17^**^	15.85	Constant	86.27^**^	15.12	Constant	108.12^**^	17.13

N = 37, ^*^ p< 0.05, ^**^ p< 0.01.

## 4 Discussion

This study explored the relationship between viewers’ mental health traits and YouTube video characteristics. We primarily considered violence, color brightness, and saturation as video characteristics, whereas viewer stress, depression, anxiety, and self-esteem were considered mental health traits. To measure violence, brightness, and saturation, we employed computer vision techniques based on deep-learning algorithms.

First, we found that the amount of violent videos consumed by an individual was positively associated with stress, depression, and anxiety, whereas it was negatively associated with viewers’ self-esteem. These results are similar to those of previous studies (21, 22). For example, Anderson et al. (21) found that stressed men watched more actions and violent TV shows, whereas Till et al. (22) discovered that individuals with depression preferred noir movies. As argued in prior studies (e.g., 22, 23), individuals with negative mental health conditions, such as stress, depression, and anxiety, might watch more violent videos to regulate their negative mental status or because such violent videos might reflect their mental status. On the other hand, the theoretical explanations about the relationship between violence in media and audience enjoyment suggest that individuals with negative mental health conditions might watch violent videos because watching violent videos generates enjoyment, which can help resolve their negative mental health status. For example, because violent scenes in media programs contain several sensory-pleasing characteristics (e.g., complexity, uncertainty, and novelty), violent scenes are likely to increase the sensory stimulation of the audience, which generates enjoyment (24, 25).

Video brightness was negatively associated with viewer stress, depression, and anxiety, which indicates that individuals with greater stress, depression, or anxiety tended to watch darker videos than those with less stress, depression, or anxiety, even though the associations were not statistically significant. These results are similar to those of previous studies (e.g., 10, 26) that found that individuals with mental health issues tended to upload darker images on social media. These studies suggest that viewers may prefer darker YouTube videos because they reflect their mental status. This result is also related to the previous finding of this study that viewers with poor mental health conditions tend to watch more violent videos on YouTube because violent videos likely contain more dark images than nonviolent videos.

On the other hand, a positive association between color saturation and mental health issues were observed, although the associations were not statistically significant. This implies that individuals with more stress, depression, or anxiety are more likely to watch videos with higher saturation. Prior studies on color saturation and emotional response (e.g., 27) suggest that more saturated colors tend to lead to higher arousal, which can in turn lead to enjoyment (28); thus, individuals with negative mental health conditions likely prefer videos with higher saturation.

This study had some limitations. The first was the small sample size of 37. The small sample size was mainly due to the limited research budget and difficulty in recruiting appropriate participants for the research. The small sample size is likely to lead to a lack of power of the research. Furthermore, to increase the degree of freedom of the statistical tests, we analyzed the partial correlation between the dependent and independent variables with some control variables (e.g., age and gender) accounted for. Second, the sample mainly comprised individuals in their 20s. Thus, the results may not be generalizable to other age groups. As discussed by some scholars (e.g., 29), violence in media programs is more likely to lead to appealing or enjoyment among young audiences than among older audiences; thus, the relationship between violence in videos and mental health traits found in this study may not be applicable to people in older age groups if violence does not lead to enjoyment in those age groups. To mitigate this limitation, the relationships should be examined for people in other age groups in the future research. Furthermore, in this study, we analyzed the videos that were featured in the watchlist of each participant. However, we were unable to determine whether the participants viewed those videos in their entirety or merely had partial exposure as YouTube did not provide such information.

In this study, we found that the characteristics of videos individuals view on YouTube varied with their mental health traits. These findings indicate that the characteristics of YouTube videos can be used to indirectly diagnose or monitor a viewer’s mental health status; such data can be used as digital phenotyping data. Diagnosing mental health problems at an early stage is difficult and individuals with mental health problems tend to avoid seeking treatment; therefore, YouTube video data can be effectively used to diagnose or monitor mental health problems. Furthermore, if data on the characteristics of videos that an individual watches on YouTube can provide additional or unique information about a user’s mental health that cannot be acquired from other types of digital phenotyping data, such as data posted on other social media platforms, then such video data can be used to more accurately assess an individual’s mental health status when combined with the other data types. We believe that the present study contributes to the field by revealing results that imply the existence of systematic relationships between video characteristics and viewers’ mental health traits, which is a significant finding when considering a vast number of YouTube users.

## Data availability statement

The raw data supporting the conclusions of this article will be made available by the authors, without undue reservation.

## Ethics statement

This study was approved by the IRB of Yonsei University, South Korea (No. 7001988-202209-HR-1672-02). The studies were conducted in accordance with the local legislation and institutional requirements. The participants provided their written informed consent to participate in this study.

## Author contributions

GC: Writing – original draft, Methodology, Formal analysis, Data curation. MP: Writing – original draft, Validation, Methodology, Data curation. S-HY: Writing – review & editing, Data curation. YK: Writing – review & editing, Methodology, Data curation. HS: Writing – review & editing, Methodology, Data curation. SL: Writing – review & editing, Writing – original draft, Project administration, Methodology, Funding acquisition, Conceptualization.

## Footnotes

1
https://roc-ng.github.io/XD-Violence/

2
https://www.kaggle.com/datasets/shreyj1729/cctv-fights-dataset

3
https://www.kaggle.com/datasets/mohamedmustafa/real-life-violence-situations-dataset

4 In the case of a video dataset, we extracted an image from a video every three seconds.
